# Enlargements of the Spleen—VIII

**Published:** 1908-09-26

**Authors:** 


### ENLARGEMENTS OF THE SPLEEN—VIII.

With Distinctive Blood Changes.

7. *Splenomegalic Polycythæmia.*—This is an affection which is being recognised with increasing frequency, and although its exact nature is not yet clear, there is little doubt that it is a definite disease. The main clinical feature is chronic livid cyanosis, coming on gradually in a patient who has previously enjoyed good health. No heart, lung, or kidney disease can be found; the spleen is variably enlarged, from being merely palpable to a mass weighing 48 ounces. The blood examination shows little or no change in the white corpuscles, either as regards total numbers or differential count, but the red cells are greatly increased—up to 9,000,000 per cub. mm. or more. There is also an increase in the hæmoglobin up to 140 or 150 per cent. of normal; in stained films nothing unusual is to be noted about the corpuscles. If a patient without any obvious lesion of any organ but the spleen suffers from chronic cyanosis, and is found to have marked polycythæmia without leucocytosis, the diagnosis of splenomegalic polycythæmia may be made with fair certainty. There are other conditions which cause polycythæmia, of course—residence in high altitudes, congenital pulmonary stenosis, chronic lung troubles, and mitral stenosis; but these seldom cause obvious enlargement of the spleen, and in any case there is the proviso that the lungs and heart must be healthy for the case to be a typical one of splenomegalic polycythæmia.

Without Blood Changes.

We may now pass on to the discussion of those enlargements of the spleen in which the blood changes are negative; in other words, in which the blood count cannot give the diagnosis at once, but merely serves to exclude the splenic conditions discussed above. The cases in this group may be roughly divided under two main headings:—

(1) Enlarged spleens with negative blood counts in children—rickets, congenital syphilis, pseudoleuchæmia infantum, splenomegalic cirrhosis.

(2) Enlarged spleens with negative blood counts in adolescents and adults—passively congested spleens, cirrhosis of the liver, splenic anæmia, Banti's disease, Hodgkin's disease.

There are two other forms of enlarged spleen that may occur at any age, namely, that of infective endocarditis and that of lardaceous disease. We will discuss these two briefly first.

*Infective Endocarditis.*—It is a remarkable fact that in ordinary cases of mechanical failure of the heart the liver usually becomes uniformly enlarged and nutmegged, but the spleen does not. One would expect that when the liver becomes overdistended with blood by passive engorgement every other organ, including the spleen, whose blood passes by the portal vein to and through the liver, would also become engorged and enlarged. This, however, is not the actual fact in the case of the spleen. In chronic heart failure it becomes engorged and dark and hard, and it may weigh a little more than usual, but its bulk seldom becomes so great that the lower pole of the organ comes definitely below the costal margin. It is rare to find a spleen of more than 10 ounces weight at an autopsy in such a case, and even then, owing to the greater hardness and density, the bulk is less than that of other 10-ounce spleens.

Quite the reverse is the case in infective endocarditis. The spleen here is nearly always to be palpated, and occasionally it extends several inches below the left costal margin. This enlargement may occur even without infarction; but infarction increases the size of the organ still more.

Infarcts in the spleen are by no means always embolic. They are sometimes thrombotic. They give rise to acute pain and tenderness in the left hypochondriac region, and a physical examination may reveal the presence of a well-marked peritoneal rub over the site. Infarcts are not proof positive of infective endocarditis, for they may occur in many different diseases; but they are highly suggestive of it. In some cases the diagnosis may be clinched by obtaining cultivations of micro-organisms from the patient's blood. It is noteworthy that infective endocarditis seldom gives rise to any decided leucocytosis.

*Lardaceous Disease.*—It is usually stated that one of the characteristics of a lardaceous spleen is that it is enlarged, but this is true only to a very limited extent. The majority of lardaceous spleens seen nowadays are scarcely bigger than the normal organ; it is only occasionally that they are sufficiently enlarged to attract particular attention. Moreover, lardaceous disease is now so rare that its importance has become comparatively slight. There are two varieties of the lesion in the spleen, one in which the change involves the Malpighian bodies only, the "sago" spleen; the other in which the blood-vessels, sinuses, and tuberculæ are affected throughout the organ, the "diffuse waxy" spleen. The patient is weak, emaciated, anæmic, and waxy looking. The liver is usually enlarged at the same time. There may be diarrhœa if the intestines are involved, and polyuria and albuminuria, with comparatively few renal tube casts in proportion to the albumen, if the kidneys are affected too. Ascites and jaundice are exceptional. The blood will show no characteristic changes, though there will be anæmia of the secondary type, with a low colour index, with or without slight leucocytosis. The diagnosis is made, first, by the exclusion of those conditions in which positive blood changes occur; and, secondly, by having a definite cause for lardaceous disease in the patient, such as long-continued suppuration, a discharging empyema sinus or hip-joint disease, or else phthisis of long standing, bronchiectasis, or well-marked syphilitic cachexia. In syphilitic cases the clinical appearance of the patient may suggest pernicious anæmia, but this is at once excluded by the colour index being found persistently less than 1.

(*To be continued.*)

